# Expression of the amyloid-β peptide in a single pair of *C*. *elegans* sensory neurons modulates the associated behavioural response

**DOI:** 10.1371/journal.pone.0217746

**Published:** 2019-05-31

**Authors:** Tessa Sinnige, Prashanth Ciryam, Samuel Casford, Christopher M. Dobson, Mario de Bono, Michele Vendruscolo

**Affiliations:** 1 Centre for Misfolding Diseases, Department of Chemistry, University of Cambridge, Cambridge, United Kingdom; 2 Cell Biology Division, Medical Research Council Laboratory of Molecular Biology, Cambridge, United Kingdom; Torrey Pines Institute for Molecular Studies, UNITED STATES

## Abstract

Although the aggregation of the amyloid-β peptide (Aβ) into amyloid fibrils is a well-established hallmark of Alzheimer’s disease, the complex mechanisms linking this process to neurodegeneration are still incompletely understood. The nematode worm *C*. *elegans* is a valuable model organism through which to study these mechanisms because of its simple nervous system and its relatively short lifespan. Standard Aβ-based *C*. *elegans* models of Alzheimer’s disease are designed to study the toxic effects of the overexpression of Aβ in the muscle or nervous systems. However, the wide variety of effects associated with the tissue-level overexpression of Aβ makes it difficult to single out and study specific cellular mechanisms related to the onset of Alzheimer’s disease. Here, to better understand how to investigate the early events affecting neuronal signalling, we created a *C*. *elegans* model expressing Aβ42, the 42-residue form of Aβ, from a single-copy gene insertion in just one pair of glutamatergic sensory neurons, the BAG neurons. In behavioural assays, we found that the Aβ42-expressing animals displayed a subtle modulation of the response to CO_2_, compared to controls. Ca^2+^ imaging revealed that the BAG neurons in young Aβ42-expressing nematodes were activated more strongly than in control animals, and that neuronal activation remained intact until old age. Taken together, our results suggest that Aβ42-expression in this very subtle model of AD is sufficient to modulate the behavioural response but not strong enough to generate significant neurotoxicity, suggesting that slightly more aggressive perturbations will enable effectively studies of the links between the modulation of a physiological response and its associated neurotoxicity.

## Introduction

Protein misfolding and aggregation is a phenomenon associated with a wide variety of neurodegenerative disorders [1–4]. In particular, in Alzheimer’s disease (AD), the most common form of dementia, amyloid-β (Aβ) and tau form characteristic deposits known as amyloid plaques and neurofibrillary tangles, respectively [5]. To understand the molecular origins of this disease, and to allow its timely diagnosis and effective treatment, it is of great importance to establish which are the mechanisms responsible for its onset and progression [6,7]. Recent evidence suggests that the appearance of amyloid plaques may precede the onset of disease symptoms by many years [8–10], and that soluble oligomeric Aβ species, rather than mature fibrils, confer the toxicity initiating synaptic dysfunction early in the disease process [11–13]. However, much remains unclear about the molecular mechanisms underlying the disease process, and no effective therapeutics are currently available.

The nematode *C*. *elegans* is a well-characterised model organism with a simple nervous system comprising 302 neurons, the connectivity of which has been fully determined [14]. *C*. *elegans* has a homologue of the human amyloid precursor protein (APP) from which the Aβ peptide is derived, but the corresponding gene (*apl-1*) does not encode for the human Aβ sequence [15]. Previously, Aβ-based *C*. *elegans* models of AD have been generated in which the human form of Aβ is overexpressed in body-wall muscle cells, leading to the formation of amyloid deposits and progressive paralysis of the worms within several days of adulthood [16,17]. Although muscle cell expression and motility phenotypes provide powerful readouts to screen for genes [18,19] or small molecules [20,21] affecting the Aβ aggregation process, neuron-specific models are particularly useful to obtain insights into the exact mechanisms linking the properties of Aβ to neurodegeneration. A strain of *C*. *elegans* has been generated with pan-neuronal overexpression of Aβ, and toxicity was inferred from defects in chemotaxis and learning, as well as a reduction in lifespan [22,23]. In another model Aβ was overexpressed in glutamatergic neurons, which comprise more than a third of all *C*. *elegans* neurons. Glutamatergic tail neurons were then inspected for signs of neurodegeneration to validate hits from a yeast genetic screen [24]. Furthermore, Cotella et al. overexpressed an Aβ construct in ASE neurons and observed chemotaxis defects [25].

In the current study, we took a different approach from traditional overexpression models, and designed a *C*. *elegans* model to investigate possible strategies for the assessment of the early events associated with Aβ expression at the single-neuron level. To accomplish this aim, we expressed the 42-residue form of human Aβ (Aβ42), the predominant component of amyloid plaques in humans, from a single-copy gene insertion in only two sensory neurons. This strategy results in more moderate expression levels than traditional plasmid overexpression, which leads to the generation of an extrachromosomal array that may contain over a 100 gene copies [26]. We chose the pair of BAG neurons, since they are glutamatergic and are thus among the neuron types most vulnerable to AD in the human brain [27–30]. The BAG neurons are tonically activated—another feature in common with human neurons vulnerable to AD [31]—by elevated concentrations of CO_2_ as well as hypoxia, and activation is linked to behavioural changes in speed and direction of travel through relatively well-understood signalling pathways [32–36]. As such, we reasoned that expression in the BAG neurons would provide clear readouts to examine Aβ42-mediated changes in neuronal function.

## Materials and methods

### *C*. *elegans* strains

Nematodes were maintained on nematode growth media (NGM) plates seeded with *Escherichia coli* OP50 at 20°C, unless stated otherwise. Strain EG6699 was maintained on NGM plates seeded with *E*. *coli* HB101 at 15°C. For age-dependent studies, a synchronized worm population was generated by a 4 h synchronized egg-lay at 20°C, after which incubation was continued at 25°C throughout the experiment unless stated otherwise.

The strains of *C*. *elegans* used in this study include:

N2 (Bristol)

EG6699 *ttTi5605* II*; unc-119(ed3)* III; *oxEx1578([eft-3p*::*GFP + Cbr-unc-119]*

AX204 *npr-1(ad609) X*

AX6171 *npr-1(ad609) dbEx[pflp-17*::*Aβ1–42*::*unc54 3’ UTR + ccRFP]*

CMD01 *ttTi5605 camIs[pflp-17*::*Aβ1–42*::*SL2mCherry*::*let-858 3’ UTR + Cbr-unc-119] II; unc-119(ed3)* III

CMD06 *ttTi5605 camIs[pflp-17*::*mCherry*::*let-858 3’UTR + Cbr-unc-119]* II; *unc-119(ed3)* III

AX2073 *dbEx[pflp-17*::*YC3*.*60]*

CMD12 *ttTi5605 camIs[pflp-17*::*Aβ1–42*::*SL2mCherry*::*let-858 3’UTR + Cbr-unc-119]* II; *unc-119(ed3)* III; *dbEx[pflp-17*::*YC3*.*60]*

CMD13 *ttTi5605 camIs[pflp-17*::*mCherry*::*let-858 3’UTR + Cbr-unc-119]* II; *unc-119(ed3)* III; *dbEx[pflp-17*::*YC3*.*60]*

CL2355 *smg-1(cc546) dvIs50 [pCL45 (snb-1*::*Aβ 1–42*::*3' UTR(long) + mtl-2*::*GFP]*

GMC101 *dvIs100 [unc-54p*::*Aβ 1–42*::*unc-54 3'-UTR + mtl-2p*::*GFP]*

### DNA cloning

DNA constructs were created using MultiSite Gateway Cloning (Thermo Fisher Scientific). As a promoter sequence, we used 2 kb upstream of *flp-17* in position 1. The sequence for human Aβ42 preceded by a signal peptide, as used previously [17], was codon-optimized for *C*. *elegans* using Jcat [37], and synthesized by GeneArt (Thermo Fischer Scientific). This construct was cloned into position 2, and the SL2::mCherry sequence in position 3. The 3’ UTR region of *let-858* was inserted into the backbone of pCFJ150 [38] in which the full construct was assembled. For the control line, mCherry was inserted in Gateway position 2 and the *let-858* 3’ UTR in position 3, followed by assembly into pCFJ150. For overexpression of Aβ42, the 3’ UTR of unc-54 was inserted into gateway position 3, and the construct with *pflp-17* in position 1 and Aβ42 with signal peptide in position 2 was assembled into pDEST. The constructs were verified by Sanger sequencing.

### Creation of transgenic strains

Strains were generated by Mos-1 single copy insertion following published protocols [38], by micro-injection of plasmid DNA into the gonads of young adults of strain EG6699. Mos-1 insertions were verified by PCR with LongAmp Taq polymerase (New England Biolabs) followed by Sanger sequencing. The Aβ42 plasmid for overexpression was micro-injected into the gonads of young adults of strain AX204 together with a plasmid encoding cc::RFP, and transgenic offspring was selected based on RFP expression in the coelomocytes.

### Antibody staining and imaging

Animals were fixed at day 3 of adulthood in 4% formaldehyde in PBS for 24 hours at 4°C, followed by β-mercaptoethanol and collagenase treatment to digest the cuticle, as described previously [39,40]. Fixed and permeabilised animals were probed with anti-amyloid-β antibody 4G8 (epitope residues 17–24), followed by Alexa-488 conjugated anti-mouse secondary antibody (both obtained from Biolegend UK Ltd). Stained animals were mounted in ProLong Gold antifade reagent with DAPI (Life Technologies) and imaged on a Leica SP8 confocal microscope using a 63x water immersion objective. To visualize the BAG neurons in living animals, the nematodes were anaesthetized with sodium azide and mounted on an agarose pad. Imaging was performed on an Andor Revolution spinning disk microscope with a 20x objective and a Leica SP8 confocal microscope using a 40x oil or a 63x oil objective.

### Lifespan assay

For lifespan assays, 150 nematodes per strain derived from a 4 h synchronized egg-lay were placed on 3 cm NGM plates containing 75 μM 5-fluoro-2’-deoxyuridine (FUDR, Sigma) at the L4 stage, and incubated at 25°C. In an independent experiment, FUDR was omitted and the nematodes were transferred daily to fresh plates during their reproductive phase. The worms were counted daily and scored as dead when they did not respond to gentle prodding with a platinum wire. Worms that crawled up against the side of the plate were excluded from the analysis. The lifespan assays were scored blindly.

### Motility assay

The motility assay was performed as described previously [41]. In brief, an age-synchronised population of animals was generated by bleaching, and worms were transferred to NGM plates containing 75 μM FUDR (Sigma) at L4 stage and incubated at 25°C. The motility assay was performed on ca. 500 animals for each strain and timepoint, and body bends per minute were determined using custom-written software.

### Behavioural assays

For behavioural assays, worms were grown on NGM plates either at room temperature (20–22°C) or at 25°C. CO_2_ conditioning was performed by raising the animals in a chamber filled with 21% O_2_ + 3% CO_2_ starting after a synchronised egg-lay. Assays on aged nematodes were performed by daily transfer to fresh NGM plates to separate the adults from their offspring. Naïve animals from the same batch that were not previously assayed were used for each time point, and at least three independent experiments were performed for each of the assays. For the CO_2_ assay, NGM plates were seeded with 20 μL of an overnight culture of *E*. *coli* OP50 approximately 20 hours before the assay. For each assay, 20 nematodes were placed onto the resulting food lawn and a 1 cm × 1cm × 200 μm polydimethylsiloxane (PDMS) chamber was placed on the plate, with gas inlets connected to a PHD 2000 Infusion syringe pump (Harvard Apparatus) as described previously [35]. The animals were allowed to adjust for several minutes before the start of the assay, followed by exposure to a 1.5 mL/min gas flow consisting of 21% O_2_ for 3 min, 21% O_2_ + 3% CO_2_ for 3 min, and finally 21% O_2_ for 3 min. For the assays of *npr-1* animals, the gases used were 7% O_2_ for 3 min, 7% O_2_ + 3% CO_2_ for 3 min, and 7% O_2_ for 3 min. Videos were recorded using FlyCapture on a Leica M165FC microscope with a Point Gray Grasshopper camera at 2 frames per second. The hypoxia assay was performed on animals starved for 4–6 h, and transferred to unseeded 3 cm NGM plates sealed with a copper ring to contain the worms within the field of view of the camera. The gas flow was applied using a custom-made setup at approximately 1–1.5 mL/min with 21% O_2_ for 6 min, 7% O_2_ for 6 min, and 21% O_2_ for 6 min. Videos were recorded using Dino-Lite Digital Microscope cameras at either 6 or 10 frames per second. Custom-written Matlab software was used to track the nematodes and to determine the fraction making omega turns, reversals and speed [35].

### Ca^2+^ imaging

Aβ42 and mCherry control lines were crossed with strain AX2073 expressing Ca^2+^ sensor YC3.60 [42] under the *flp-17* promoter, driving expression in BAG. The assay was performed on freely moving animals using the set-up described previously [43]. Individual animals were placed on 5 cm agarose plates (17 g/L agarose, 3 g/L NaCl, 5 mg/L cholesterol, 1 mM MgSO4, 1 mM CaCl2) seeded with a 3 μL concentrated drop of *E*. *coli* OP50. A PDMS chamber was placed on top and the gases were applied at 1.4 mL/min, with 21% O_2_ for 2 min, 21% O_2_ + 3% CO_2_ for 2 min, and finally 21% O_2_ for 2 min.

Videos were recorded at 10 frames per second using 100 ms exposure time on a Nikon AZ100 microscope with an AZ Plan Fluor 2x lens. A TwinCam adaptor (Cairn Research, UK) and two ORCA-Flash4.0 V2 digital cameras (Hamamatsu, Japan) were used to simultaneously record YFP and CFP fluorescence. The neurons were tracked and the YFP/CFP ratios (R) calculated using custom-written Matlab software. R_0_ was defined as the initial value averaged over the first 100 frames (10 s) and ΔR/R_0_ expressed as the percentage increase.

### X-34 staining

Animals were grown at 25°C until day 7 of adulthood, washed in M9 buffer and incubated in 1 mM X-34 (Sigma) in 10 mM Tris-HCl pH 8.0 at room temperature for 3 h. They were then transferred to seeded NGM plates and allowed to destain for ca. 16 h. Imaging of anaesthetised animals was performed as described above on a Leica SP8 with 63x oil objective.

### Statistics

A Mann-Whitney U-test was applied for statistical analysis of the omega turn response over the time intervals as indicated in the figures. A *t*-test was used to examine speed, unless the data were not normally distributed in which case the Mann-Whitney U-test was applied. Ca^2+^ levels were assessed with a t-test over the interval of interest. The data were considered statistically significant at *p* < 0.05.

## Results and discussion

### A *C*. *elegans* model expressing Aβ42 in the two BAG neurons (BAG-Aβ worms)

**Fig 1:**
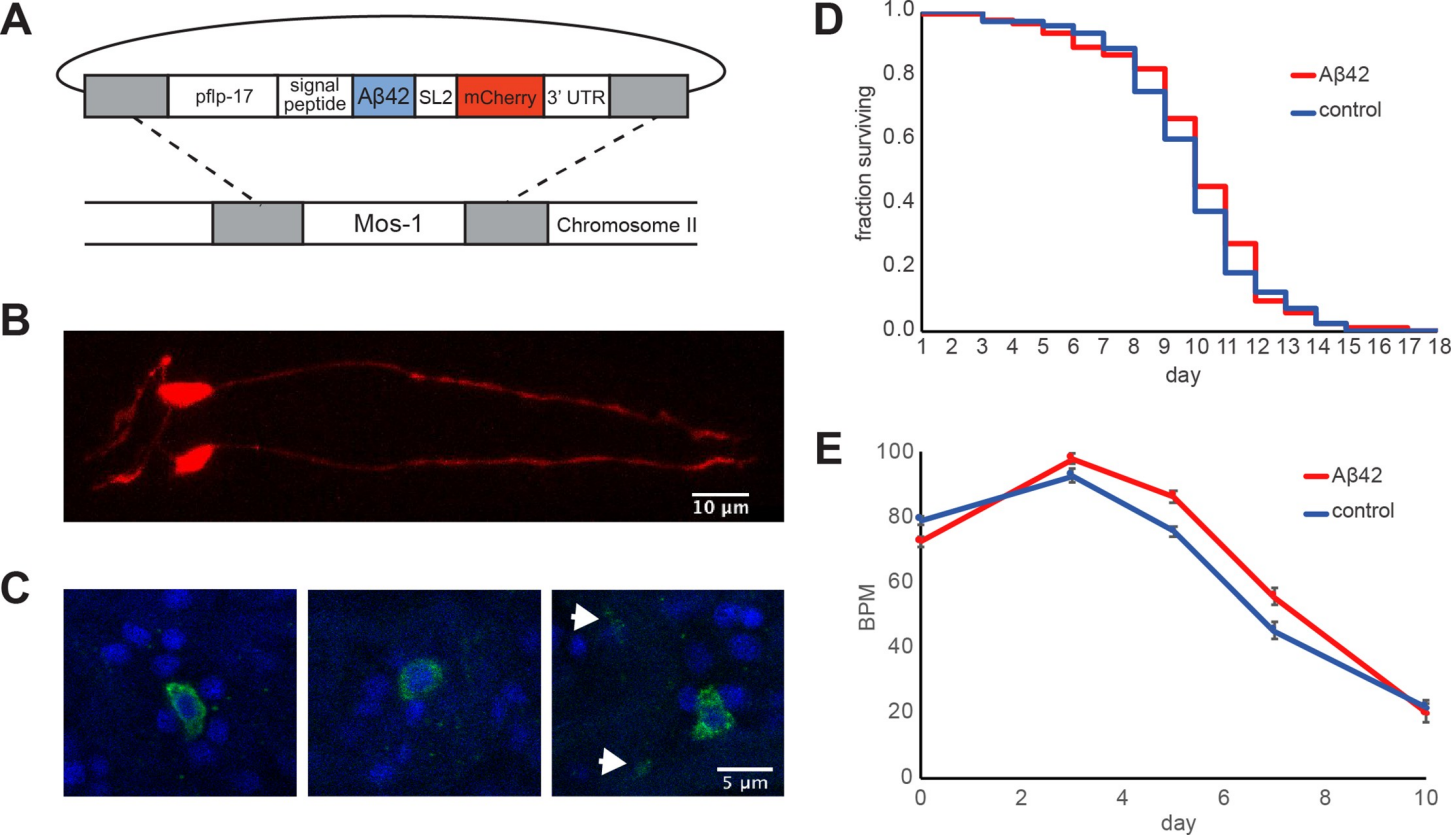
Construction of a *C*. *elegans* model that expresses Aβ42 in the two BAG neurons. (A) Design of the construct for Mos1 insertion. (B) Confocal image of the Aβ42 strain showing mCherry expression in the BAG neurons. (C) Staining with the 4G8 anti-Aβ (17–24) antibody (green), which shows that Aβ is localized primarily around the nuclei (visualized by DAPI staining, blue). In some cases, weak staining was observed in a region that may correspond to the BAG axons (white arrows). (D) Lifespan assay. The worms were scored daily in response to gentle prodding with a platinum wire, and the number of animals that remained alive was plotted as a fraction of the number of L4 worms at the start of the experiment. The assay was performed in a blinded fashion and two independent experiments showed no difference in lifespan between Aβ42 and control strains. (E) Motility assay. Body bends per minute are plotted for BAG-Aβ and control strains, averaged over two independent experiments. Error bars indicate standard error of the mean.

We designed a construct encoding human Aβ42 (codon-optimized for *C*. *elegans*) targeted to the secretory pathway by a signal peptide, an approach which was previously employed for Aβ overexpression lines and results in the 42-residue Aβ peptide after cleavage of the signal sequence [17]. We used the *flp-17* promoter to induce robust expression exclusively in the pair of BAG neurons. As a marker for expression, we incorporated mCherry after an SL2 trans-splice site, making an operon that separately expresses cytoplasmic mCherry. To ensure reproducible expression at relatively low levels, we generated integrated lines using Mos1 single copy insertion (Fig 1A) [38].

We observed mCherry expression throughout the BAG neurons (Fig 1B), whereas antibody staining indicated that the expressed Aβ42 mainly resided intracellularly, being visible surrounding the nucleus (Fig 1C). Although our Aβ42 construct comprises a signal sequence that was originally designed for secretion [16], it has been demonstrated previously that cleaved Aβ42 accumulates intracellularly when expressed in body wall muscle cells, and our findings are thus in line with the suggestion that Aβ42 may be unable to exit the secretory system in these models [16,44].

In addition, we generated a control line expressing only cytoplasmic mCherry in the BAG neurons, and performed life span assays to characterise the model when grown at 25°C, the temperature that was previously shown to maximise toxicity in a strain overexpressing Aβ42 in the body wall muscle cells [17]. We found that the lifespan of both strains was virtually identical with a median of 10.9 days for the animals expressing Aβ42, compared to 10.8 days for the control animals (Fig 1D). In a motility assay, the BAG-Aβ worms did not show impairments either compared to control animals throughout their lifespan (Fig 1E). These results indicate that Aβ42 expression in the BAG neurons did not cause widespread toxic effects in our model, in contrast to the reduced lifespan reported for a strain with pan-neuronal Aβ42 overexpression [23] and the strong paralysis observed for the muscle model [17].

### Modulation of the behavioural response of the BAG-Aβ worms

**Fig 2:**
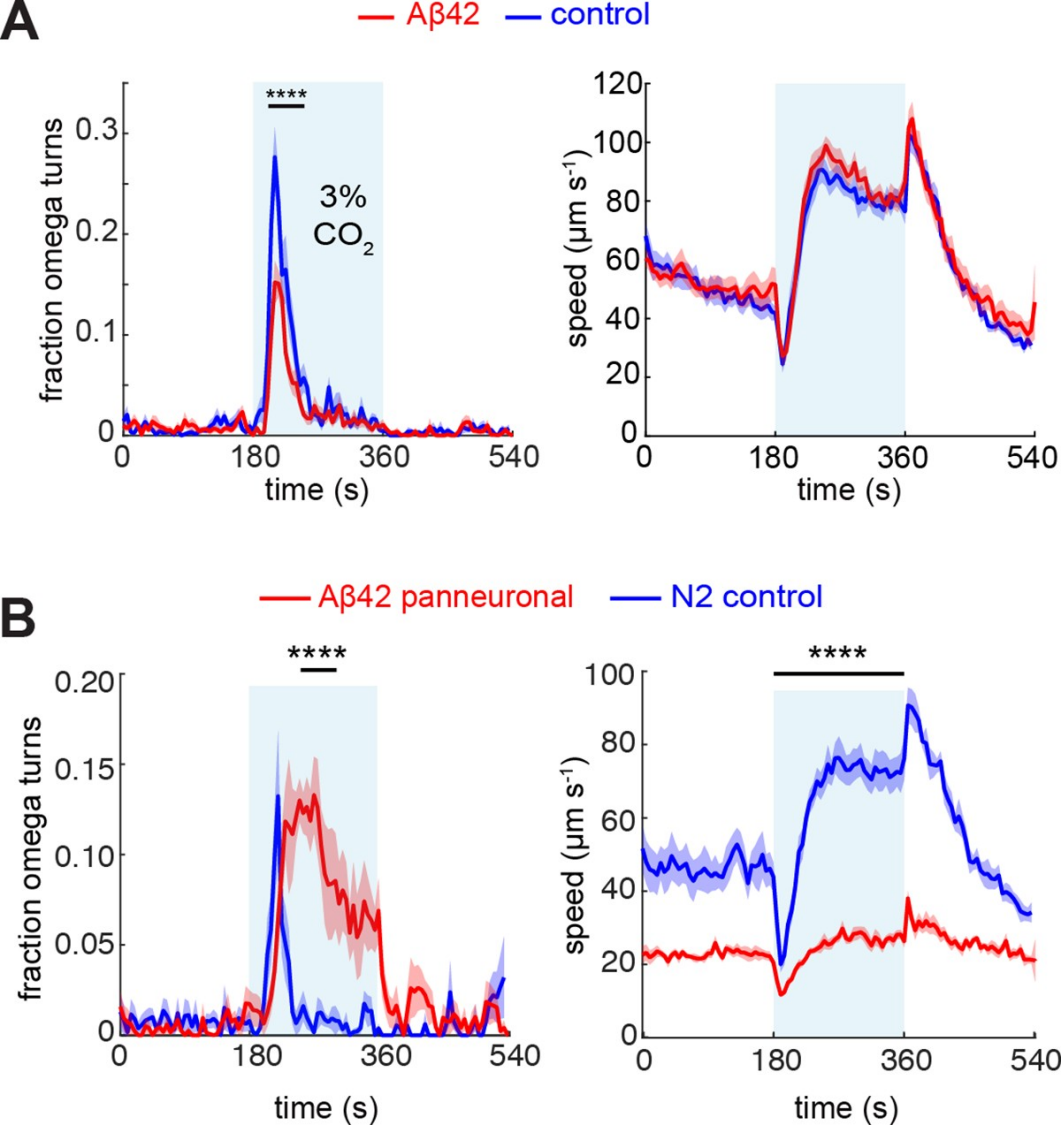
CO_2_ assay of Aβ-expressing strains. (A) Fraction of omega turns (top) and speed (bottom) of BAG-Aβ worms (red, n = 221) and mCherry controls (blue, n = 228) in response to 3% CO_2_. (B) CO_2_ assay of strain CL2355 with panneuronal Aβ-overexpression (red, n = 172) and N2 controls (blue, n = 170). Statistical tests were performed with a Mann-Whitney U-test, **** *p* < 0.0001.

We then set out to examine the effects of Aβ42 on the function of the BAG neurons using behavioural assays. The BAG neurons mediate *C*. *elegans* avoidance of elevated concentrations of CO_2_, via a transmembrane guanylate cyclase which signals opening of the TAX-2/TAX-4 cGMP-gated ion channel [33,34]. At the behavioural level, BAG activation induces characteristic omega-shaped turns of the animals in response to a CO_2_ stimulus. In the behavioural assay, animals were allowed to crawl on a seeded nematode growth media (NGM) plate, and covered by a microfluidic chamber to which the desired gas mixtures were applied [35]. We used a custom-written software to track the worms and automatically determine their speed and turning, as described previously [35]. In this assay, we observed a reduction in the fraction of Aβ42 animals making omega turns with respect to controls, whereas speed was unaffected (Fig 2A). We confirmed that this phenotype was associated with Aβ42-expression, rather than resulting from an undesired mutation that may have occurred while creating the transgenic strain, in an independent experiment on animals overexpressing Aβ42 in BAG. This strain was created in an *npr-1* mutant background which requires the CO_2_ assay to be performed at 7% O_2_, and we observed that also under these conditions Aβ42-expression was associated with a reduction in omega turns (S1 Fig). In contrast, subjecting the strain overexpressing Aβ throughout the nervous system [22] to the CO_2_ assay resulted in a different phenotype. L4 animals were incubated at 25°C overnight prior to the assay which is required to induce Aβ expression in this model, and the assayed adults showed a prolonged period of omega turns and a strong reduction in overall speed compared to wild-type N2 worms treated in the same way (Fig 2B). This phenotype is likely caused by several types of neurons being affected by Aβ-overexpression in this strain, including interneurons and motor neurons, demonstrating the importance of neuron-specific expression to examine BAG-related behavioural changes.

**Fig 3:**
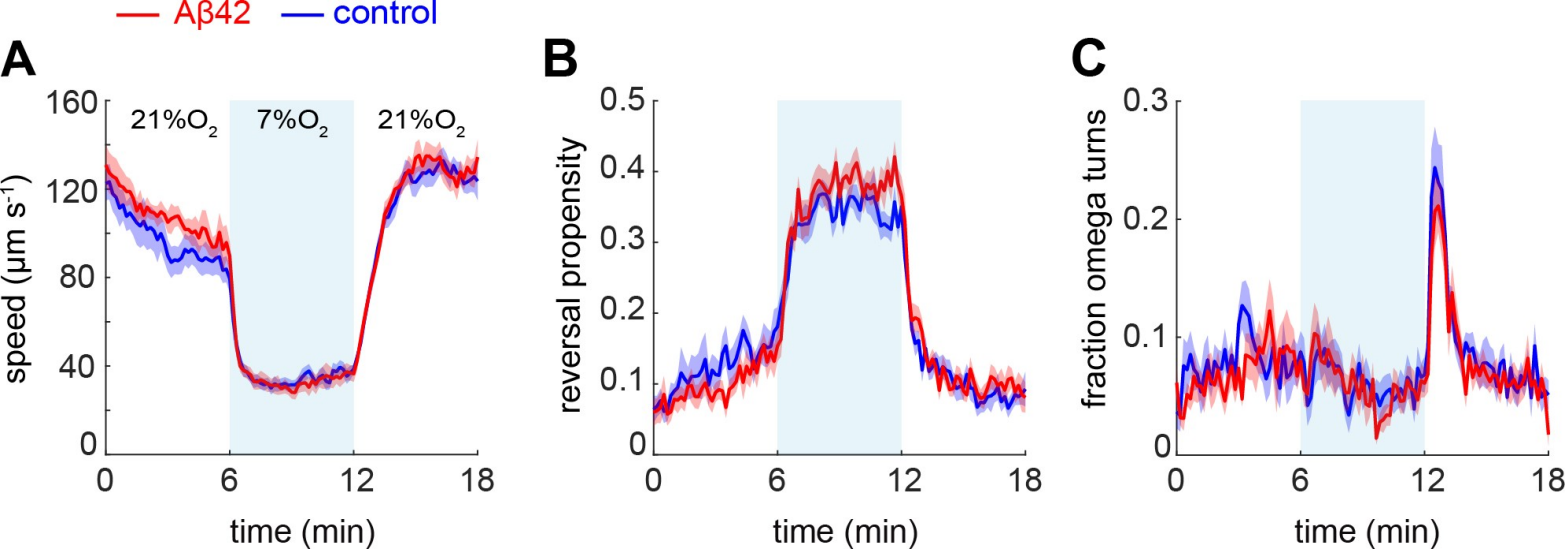
Hypoxia assay of starved BAG-Aβ and mCherry control animals. (A) Speed response, (B) reversal propensity, and (C) fraction of omega turns of BAG-Aβ (red, n = 201) and control (blue, n = 209) animals in response to a downshift from 21% to 7% O_2_ and upshift back to 21% O_2_.

To further characterise the behavioural phenotype of the BAG-Aβ worms, we employed another assay that depends on BAG function, in which starved animals respond to a decrease in oxygen concentration by reducing their speed [45] (Fig 3). We observed that BAG-Aβ and control animals similarly slowed down (Fig 3A) and increased their reversals (Fig 3B) upon a downshift of 21% to 7% O_2_, both of which responses were previously shown to be abolished in BAG-ablated animals [45]. Furthermore, we found a peak in the fraction of animals making omega turns when the concentration was upshifted from 7% to 21% O_2_. Although the maximum omega turn response in the Aβ-expressing worms remained below that of the controls, this effect did not reach statistical significance (Fig 3C).

**Fig 4:**
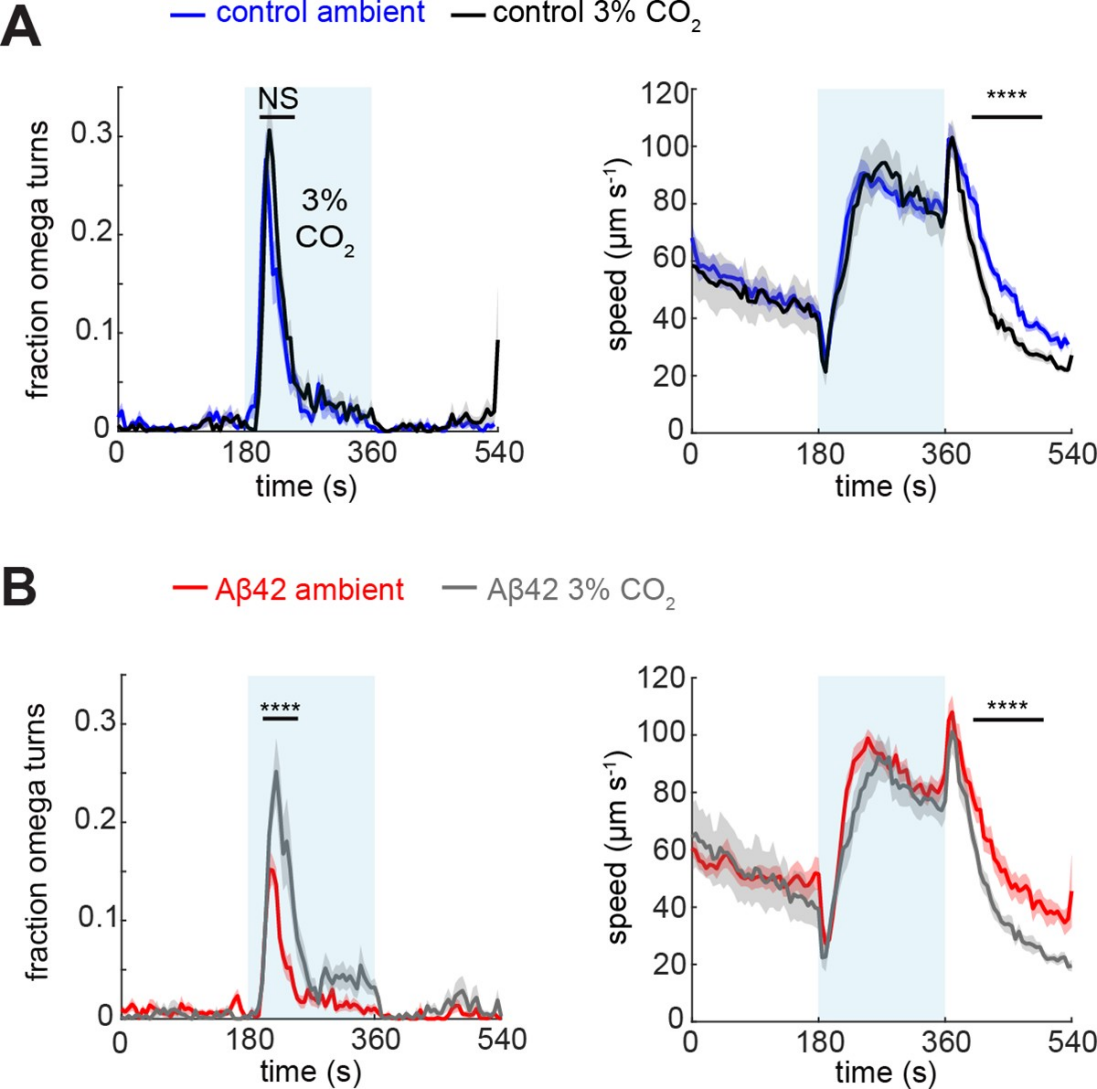
Experience-dependent modulation of the CO_2_ response. Behavioural response of (A) control animals raised at ambient atmosphere (blue, n = 228) or at 3% CO_2_ (black, n = 216), (B) BAG-Aβ animals raised at ambient atmosphere (red, n = 221) or or at 3% CO_2_ (grey, n = 215). Omega turns were assessed using a Mann-Whitney U-test and speed with a *t*-test over the indicated intervals. **** *p* < 0.0001.

Next, we were interested in exploring the effects of Aβ42 expression on BAG-dependent learning and memory. The BAG neurons were recently shown to mediate experience-dependent changes in chemotaxis behaviour towards CO_2_ [46]. When we raised the nematodes at an elevated CO_2_ concentration of 3%, we observed in our CO_2_ assay that control animals did not change their omega turn propensity (Fig 4A, left), but had a steeper decrease in speed after removal of the CO_2_ stimulus (Fig 4A, right). In BAG-Aβ worms, on the other hand, the fraction of omega turns was increased upon CO_2_ conditioning (Fig 4B, left), suggesting they had become sensitised. The modulation of the speed decline after the stimulus was similar to that of controls (compare right panels of Fig 4B and Fig 4A), indicating that Aβ42 expression did not abolish experience-dependent modulation of the behavioural response.

### Behavioural response, but not neuronal activation, declines with age

**Fig 5:**
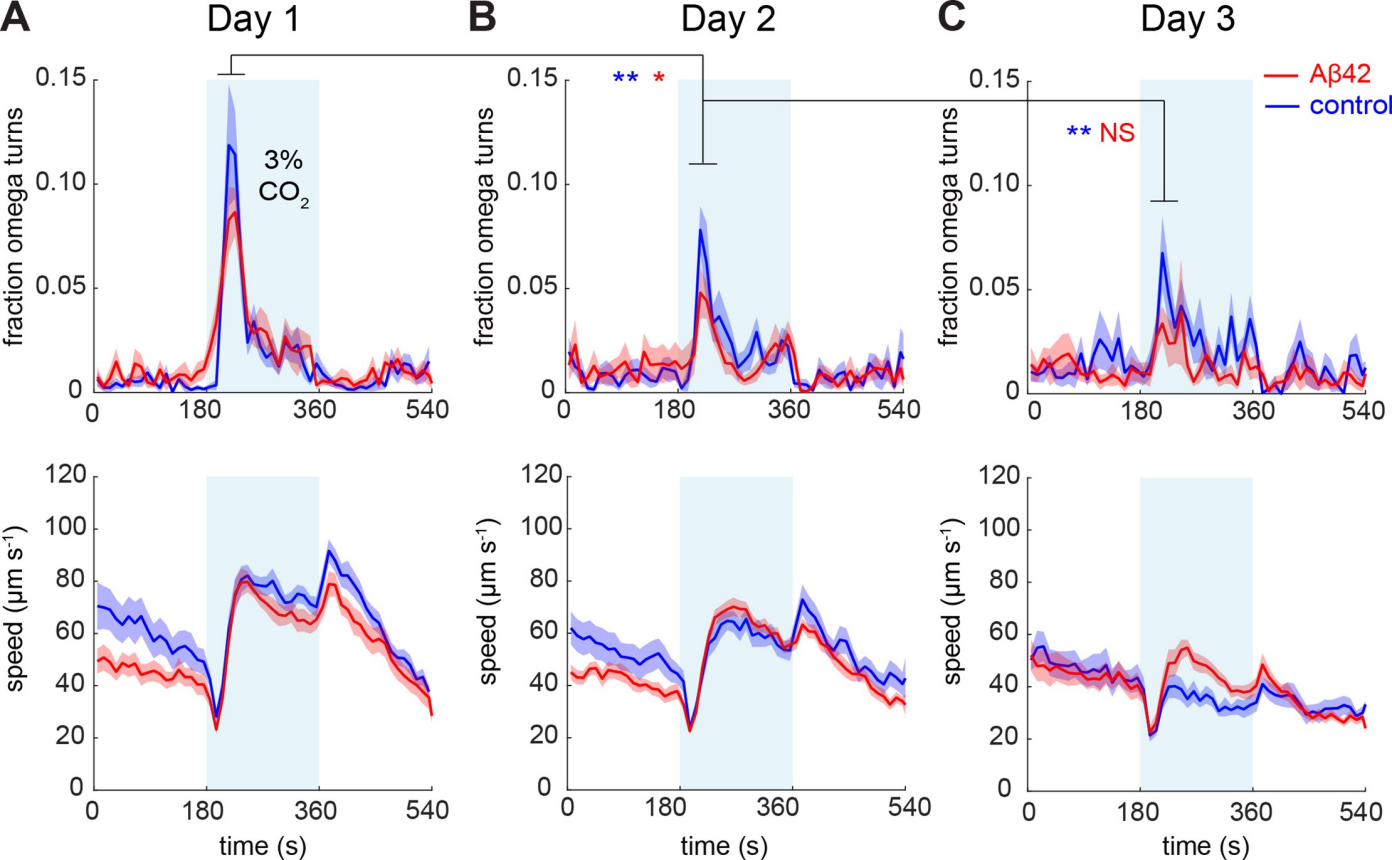
Locomotory responses to 3% CO_2_ as a function of ageing. Shown are the fraction of animals making omega turns (upper panels) and the modulation of speed (lower panels) in response to a 3% CO_2_ stimulus (shaded in blue) at day 1 (A), day 2 (B) and day 3 (C) of adulthood. For each time point, naïve animals not previously assayed were taken from the same batch of animals. The data shown here were averaged over multiple independent experiments, Aβ n = 220–280, control n = 160–241. Statistics on the fraction of omega turns were performed with a Mann-Whitney U-test over the indicated time intervals. * *p* < 0.05, ** *p* < 0.01, NS not significant.

We continued to examine the effects of ageing on the Aβ42-associated phenotype in our neuron-specific model by assaying the animals at day 1, 2 and day 3 of adulthood, when raised at 25°C (Fig 5). We found that the difference between Aβ42 and control animals in response to CO_2_ did not increase with age, and that omega turns in both lines strongly declined with age (Fig 5). A similar trend with age was observed for animals raised at 20°C, as well as for the N2 wild-type laboratory strain, indicating that this effect was not caused by mCherry expression or by the elevated growth temperature (S2 Fig).

By contrast, the typical BAG-mediated ‘off-response’, observed as a transient increase in speed directly after switching off the CO_2_ stimulus [36], remained intact at all ages examined (Fig 5 and S2 Fig lower panels). We did not, however, observe a consistent difference between Aβ42 and control animals using speed as a read-out. The use of this parameter was furthermore limited due to the age-associated overall reduction in speed, which may reflect muscle decline rather than neuronal malfunction [47].

**Fig 6:**
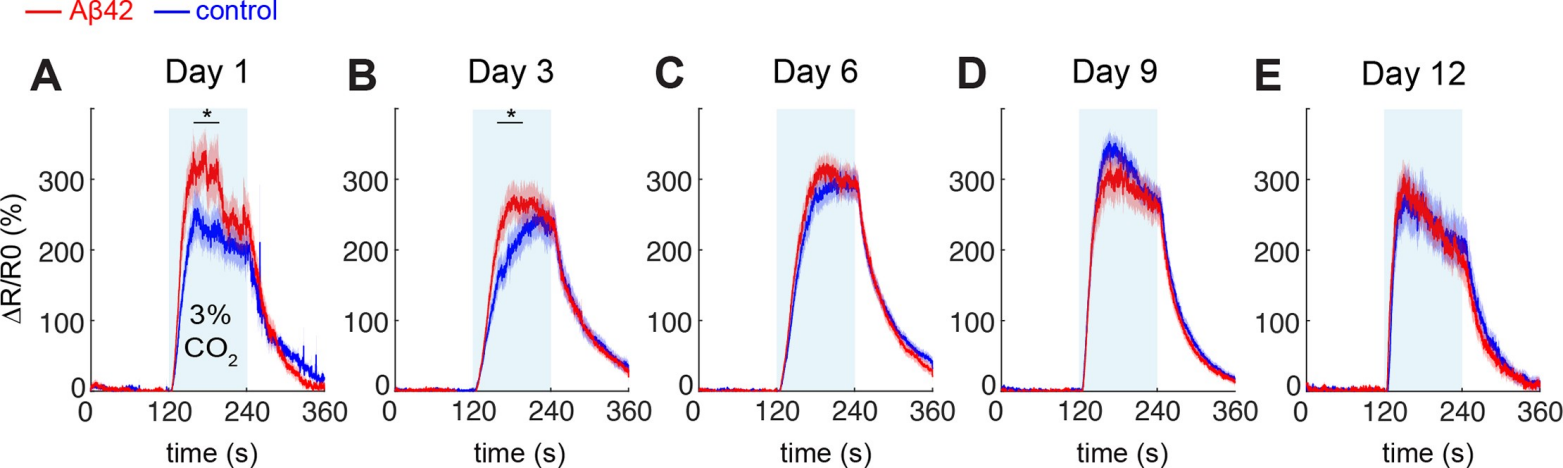
Ca^2+^ response in the Aβ-BAG worms and corresponding controls. (A) Day 1 of adulthood, (B) day 3, (C) day 6, (D) day 9, (E) day 12. For each measurement, naïve animals not previously exposed to elevated CO_2_ levels were taken from the same batch of age-synchronised animals. Data were averaged over 2–4 biological replicates for each time point, n = 23–28 per strain for day 1, 3, 6, 9 and n = 11–13 for day 12. ΔR/R_0_ represents the percent increase in YFP/CFP ratio, normalized to the baseline value. Statistics were performed using a *t*-test between 160 s and 200 s, * *p* < 0.05.

We next turned to Ca^2+^ imaging as a way to directly assess the response of the BAG neurons. The Förster resonance energy transfer (FRET) sensor YC3.60 [42] was expressed in the BAG neurons, and we monitored the increase in intracellular Ca^2+^ concentration upon activation with 3% CO_2_ (Fig 6). We found that there was a significantly elevated Ca^2+^ response in young Aβ42-expressing worms compared to controls (day 1 *p* = 0.04, day 3 *p* = 0.03) (Fig 6A and 6B), and that this difference disappeared in older animals (Fig 6C–6E). Furthermore, we found that the CO_2_-evoked Ca^2+^ response in BAG remained intact as the animals aged, showing no signs of impairment as a result of Aβ42 expression or age-related effects. Even at day 12, beyond the median lifespan of 11 days, the Ca^2+^ response was preserved (Fig 6E), suggesting that the BAG neurons remain functional at least at the level of Ca^2+^ activation throughout the lifespan of *C*. *elegans*.

**Fig 7:**
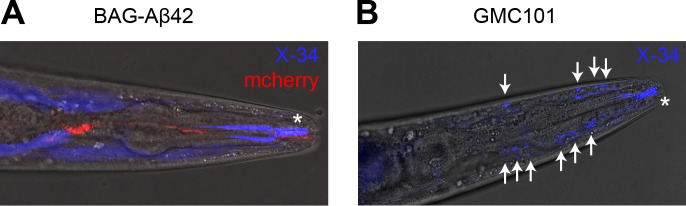
Staining with X-34 does not reveal amyloid deposits in BAG-Aβ42 worms. (A) BAG- Aβ42 animal grown until day 7 at 25°C followed by live staining with X-34. X-34 staining (blue) is observed diffusely throughout the body and at the mouth cavity (asterisk), but not observed to co-localise with the BAG neurons (red). (B) GMC101 animal overexpressing Aβ42 in the body wall muscle cells grown until day 7 at 25°C, showing deposits positive for X-34 (blue, deposits marked with arrows).

Consistent with the modest phenotype of the BAG-Aβ42 worms, and the absence of neurodegeneration as judged from the Ca^2+^ response, we failed to detect amyloid deposits using the amyloid-binding dye X-34 (Fig 7). However, the presence of soluble oligomeric species cannot be ruled out and may be responsible for the behavioural phenotype and the increased Ca^2+^ activation observed in young adults. It is interesting to note that neuronal hyperactivation is thought to occur in the early stages of human AD, prior to the appearance of clinical symptoms [48].

## Conclusions

We have reported the creation and characterisation of a *C*. *elegans* model expressing human Aβ42 in the BAG neurons from a single gene copy. Whereas *C*. *elegans* models based on tissue-wide overexpression of Aβ tend to exhibit toxicity in terms of paralysis, shortened lifespan, chemotaxis and memory defects, our model appears to remain generally healthy. We did, however, find specific alterations of the behavioural responses mediated by the BAG neurons. These changes may reflect perturbations in neuronal signalling mediated by soluble Aβ42 species and which may be relevant to the early stages of AD. Alternatively, the ectopic expression of human Aβ42 may modulate synaptic transmission and plasticity in young *C*. *elegans* animals as part of its physiological role [49]. Overall, these results suggest that effective strategies to create animal models of Alzheimer’s disease could be developed by generating perturbations sufficient to modulate specific physiological responses, but not strong enough to cause directly more widespread toxicity processes. Subjecting such models to additional perturbations or forms of stress may be sufficient to trigger the disease process in a more physiological manner than in traditional overexpression models.

## Supporting information

S1 Fig Behavioural response of animals overexpressing Aβ42 in BAG in *npr-1* background. CO_2_ response of *npr-1* animals overexpressing Aβ42 in BAG (red, n = 280) and non-transgenic siblings (blue, n = 89) in 7% O_2_ background.
(TIF)

S2 Fig Behavioural response of the BAG-Aβ worms and N2 wild-type worms raised at 20°C. A-C) The fractions of worms making omega turns in response to 3% CO_2_ (shaded in blue) at day 1 (A), day 2 (B) and day 4 (C) of adulthood. D-E) Speed response at day 1 (D), day 2 (E) and day 4 (F). The data represent 2–3 assays for each time point and strain from one biological replicate, Aβ n = 66 (day 1), n = 71 (day 2), n = 47 (day 4); N2 n = 46 (day 1), n = 66 (day 2), n = 65 (day 4).
(TIF)
