# Supplementary figures and images for: Expression of the amyloid-β peptide in a single pair of C. elegans sensory neurons modulates the associated behavioural response
Source: PLoS One. 2019 May 31;14(5):e0217746. doi: 10.1371/journal.pone.0217746 (PMC6544271; doi:10.1371/journal.pone.0217746)

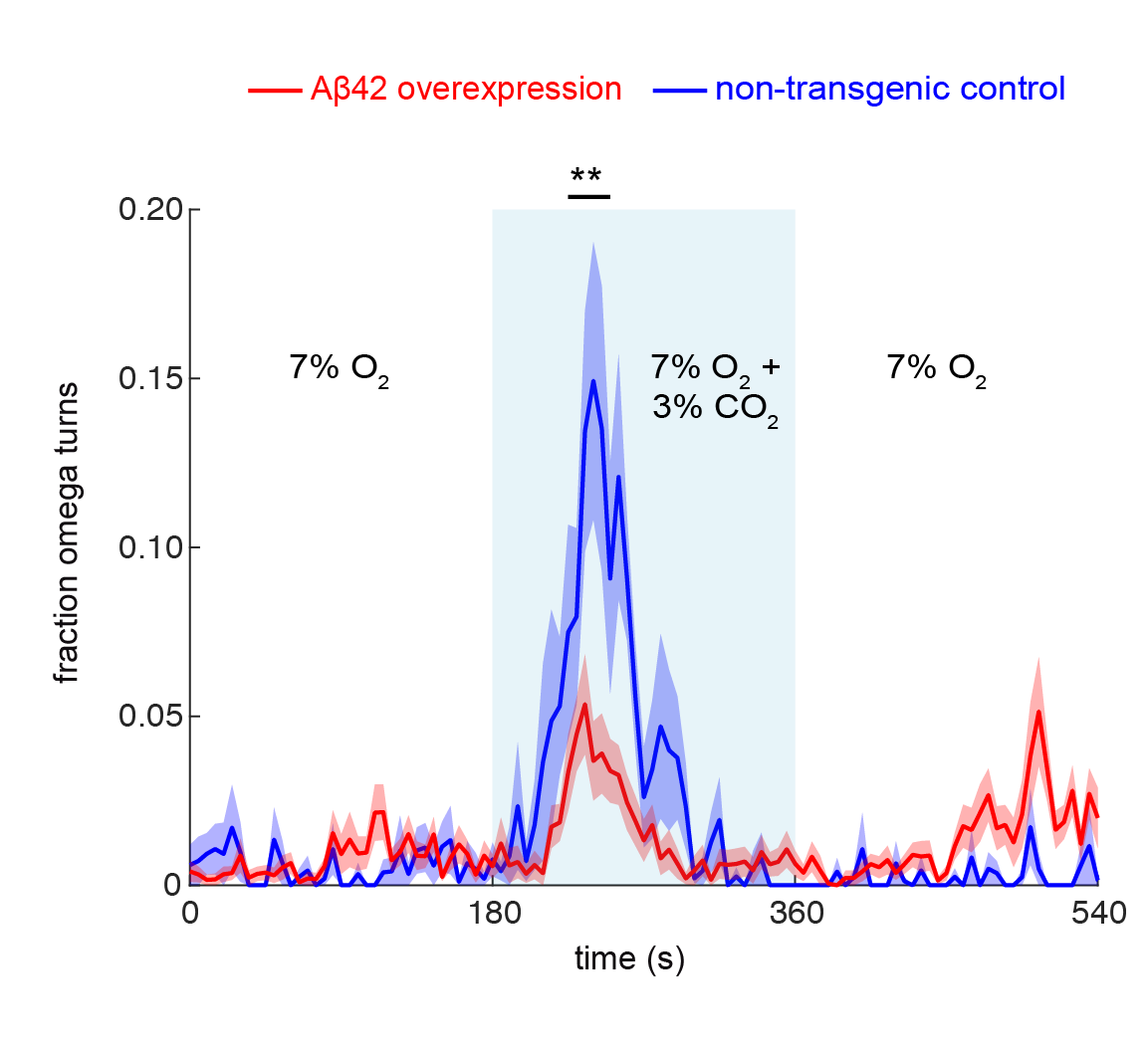

Supplement: S1 Fig — CO2 response of npr-1 animals overexpressing Aβ42 in BAG (red, n = 280) and non-transgenic siblings (blue, n = 89) in 7% O2 background. (TIF) [file pone.0217746.s001.tif]

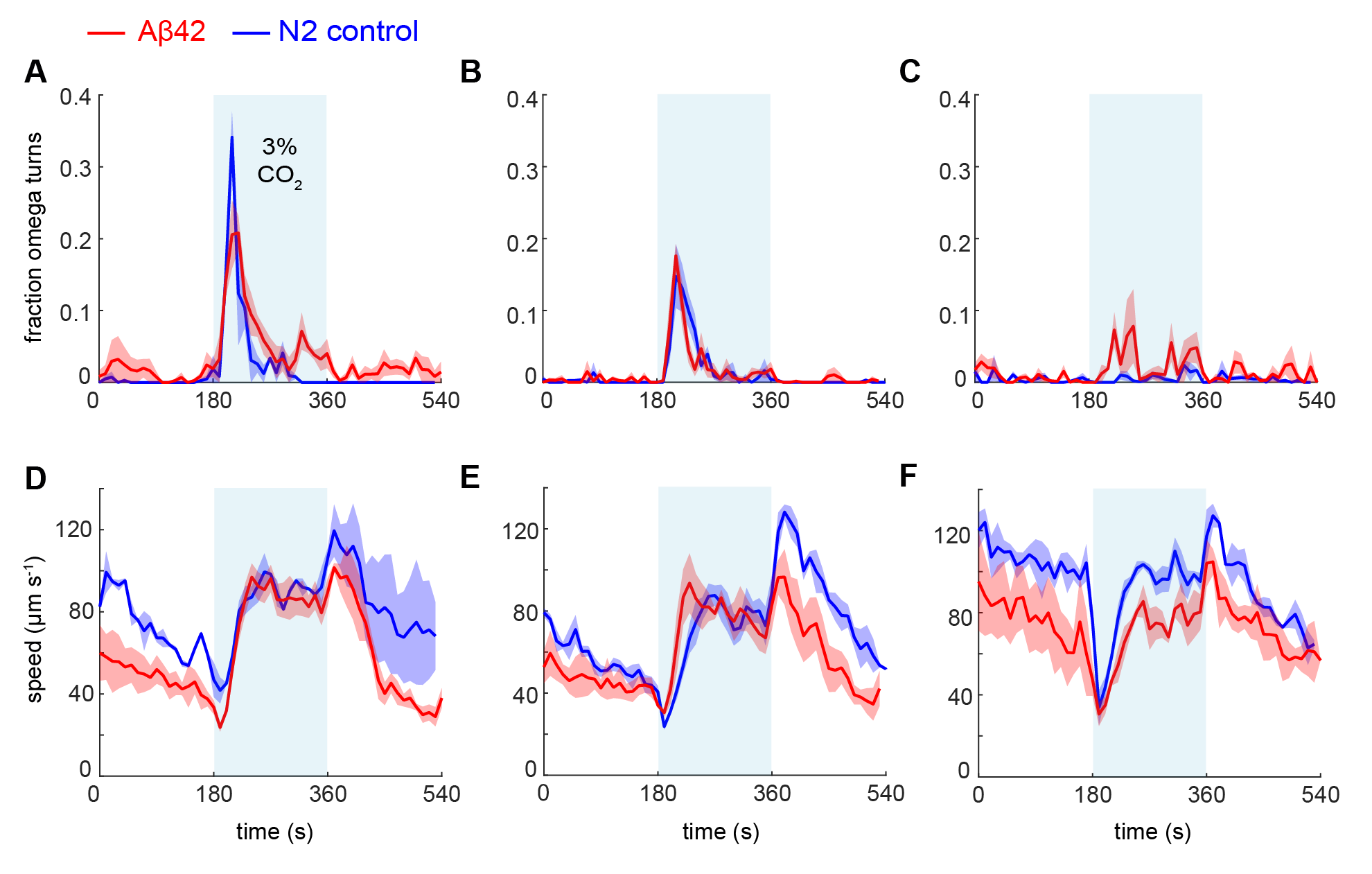

Supplement: S2 Fig — A-C) The fractions of worms making omega turns in response to 3% CO2 (shaded in blue) at day 1 (A), day 2 (B) and day 4 (C) of adulthood. D-E) Speed response at day 1 (D), day 2 (E) and day 4 (F). The data represent 2–3 assays for each time point and strain from one biological replicate, Aβ n = 66 (day 1), n = 71 (day 2), n = 47 (day 4); N2 n = 46 (day 1), n = 66 (day 2), n = 65 (day 4). (TIF) [file pone.0217746.s002.tif]
